# A 2-Gbps low-SWaP quantum random number generator with photonic integrated circuits for satellite applications

**DOI:** 10.1038/s41534-025-01100-2

**Published:** 2025-09-26

**Authors:** Oliver M. Crampton, Toby J. Dowling, Thomas Roger, Peter R. Smith, James F. Dynes, Matthew S. Winnel, Davide G. Marangon, Mirko Sanzaro, Ravinder Singh, Chithrabhanu Perumangatt, Joseph A. Dolphin, Taofiq K. Paraiso, Andrew J. Shields

**Affiliations:** 1 Toshiba Europe Limited, Cambridge, UK; 2 School of Engineering and Physical Sciences, Heriot-Watt University, Edinburgh, UK

**Keywords:** Fibre optics and optical communications, Quantum information

## Abstract

We introduce a low size, weight and power quantum random number generator (QRNG) utilizing compact integrated photonic asymmetric Mach-Zehnder interferometers (AMZIs). Our QRNG is based on phase-diffusion in two gain-switched lasers interfered within two separate chip-AMZIs. By substituting the high-bit analog-to-digital converters, typically employed to digitize the random intensity signal from each laser, with clocked comparators we significantly reduce both the complexity and power consumption of the device. Furthermore, by performing the exclusive OR (XOR) operation on the output random bits of each channel we are able to reduce the processing requirements. The QRNG architecture can be integrated with an overhead power consumption of just 7.93 W, accounting for the opto-electronics and FPGA implementation, providing fast random number generation at up to 2 Gbps. We demonstrate the real-time seeding of a free-space decoy-state quantum key distribution system using our QRNG. Our design and implementation provides a practical solution for QRNGs requiring low-power and high bit rates. This advancement is important for practical QRNGs and particularly for application in resource-constrained environments such as space-based quantum key distribution.

## Introduction

Advancements in quantum computing^1^ have cast doubt on the safety of public key infrastructure. While quantum key distribution (QKD) promises to solve this problem, providing information theoretic security^2^, the technology relies on a source of unpredictable random numbers to generate the key^3^. In recent years, quantum communications systems have seen an increase in clock rate, and are now typically operated in the gigahertz regime. For QKD systems that are required to operate in constrained environments, such as on board satellites^4^ or in isolated or difficult to access locations, the size, weight and power (SWaP) needs to be carefully considered. Therefore, there is a need for low-SWaP, high-speed and high quality random number generators.

Random number generators which exploit physical processes that result in unpredictable outcomes as a source of entropy are classed as true random number generators (TRNGs). Examples of such processes include thermal^5^ and electronic noise^6^. A subset of TRNGs, known as quantum random number generators (QRNGs), generate unpredictable random numbers based on the outcome of intrinsically random quantum mechanical processes, such as quantum superposition^7^ or entanglement^8^.

One of the simplest quantum systems proposed as a QRNG is single photons incident to a beamsplitter^9^. However, the bit-rate of these QRNGs is limited to Mbps by the detector dead-times^7,10^. The random numbers used by modern high-rate QKD systems, operating at GHz clock rates, need to be generated at multiple Gbps^11^, making these lower generation rate QRNGs unsuitable. Significantly higher bit rates have been achieved with vacuum^12,13^, intensity^14^ and phase noise^15^ approaches. These QRNGs vary significantly in their implementation, however, all such systems face the same challenges in resource constrained environments, for example, satellite QKD transmitters. To enable this, four main challenges must be overcome; the size, weight, power and generation rate.

Recent advancements in photonic integration and nano-fabrication are addressing the size and weight^16,17^, making all types of quantum architectures more compact. Another challenge comes from the limited-power supply, typically 5–22 W for small CubeSats^18^. High generation rates are typically achieved by using high bit-depth analog-to-digital converters (ADCs) which consume relatively large amounts of power, typically 2.5–4.5 W per device. Furthermore, the power consumption of the complex algorithms used to extract uniformly distributed, unpredictable random numbers from the raw outputs of QRNGs is also high^19^. Therefore, in this context, alternative approaches must be considered.

These issues are overcome by the QRNG architecture we propose. We use compact photonic integrated asymmetric Mach-Zehnder interferometers (AMZIs) and low power laser diodes as gain switched randomness sources. We also introduce a clocked comparator^20^, replacing the multi-bit ADCs commonly used in most similar architectures. In our device, two independent channels are employed to generate random outputs, these are combined by performing the exclusive OR operation bit-wise to obtain a sequence of uniformly distributed random numbers. Our modifications to the electronic design reduce the power consumption on the PCB by up to 40% compared to using a multi-gigasample per second 8-bit ADC.

In this article, we present a low-SWaP QRNG based on spontaneous emission phase noise in laser diodes (LDs). The system can output random numbers at up to 2 Gbps. It is worth mentioning that, similar to QKD, QRNGs also have security frameworks, classified based on the assumptions underlying the protocols. These frameworks include the device-independent^21,22^, semi-device-independent^23,24^, and device-dependent models. The first two offer the highest levels of assurance, as they assume that an adversary may have full or partial control, respectively, over the QRNG. However, this enhanced security comes at the cost of complex setups and/or limited generation rates. As explained, to ensure compatibility with satellite operations, we prioritized simplicity and speed, making the device-dependent framework a natural fit for the low-SWaP QRNG. In our case, assurance is provided by the use of a well-known physical process widely employed in QRNGs—namely, the interference of phase-randomized fields—along with an in-depth characterization of the hardware setup and its optimal operating parameters, as will be shown in the next section. In fact, we first characterize the phase-randomization of our lasers, then demonstrate gain-switched operation is stable over long acquisition times, and pass the NIST statistical randomness test suite. Finally we demonstrate the direct applicability of our Low-SWAP QRNG to quantum secure communications by supplying random numbers, in real-time, to a free-space QKD system operating a decoy-state BB84 protocol over a 20 dB-loss channel.

## Results

### Optical characterization of QRNG

**Fig. 1:**
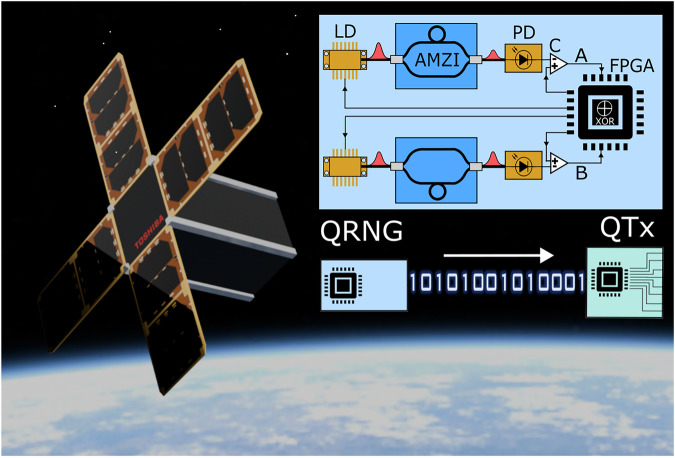
Low-SWaP QRNG. Top-right, a schematic of the QRNG design, including two gain-switched DFB lasers (LD) each connected to an integrated photonic AMZI and photodiode (PD). Both channels, A and B, are shown. The XOR is implemented on the FPGA. Bottom-right Demonstration of our QRNG being used to seed the quantum transmitter (QTx) for QKD. Left, a potential CubeSat application for our Low SWaP QRNG.

The scheme for our QRNG is shown in Fig. 1, along with the simplified integration of the QRNG output bit-stream into the QKD system. Our low-SWaP QRNG is based on the interference of phase-randomized pulses^25,26^. In this approach, two independent distributed feedback laser diodes (DFB lasers, 1550 nm) are periodically driven above and below their lasing threshold. This process, known as gain-switching, produces trains of pulses whose phases are randomized due to spontaneous emission phase noise which is dominant below lasing threshold^27^.

When these phase-randomized pulses pass through their respective asymmetric Mach-Zehnder interferometers (AMZIs) with fixed 1 ns delay (see “Methods”), they interfere with time-delayed copies of themselves. Because each pulse interferes with the previous one and the pulse phases are uniformly random, the phase differences between consecutive pulses are also uniformly random. This random interference leads to intensity fluctuations at the output of the AMZIs, with the probability of observing particular intensity values following the characteristic arcsine distribution. The intensity is recorded by photodiodes, and clocked comparators convert the analog signals into digital bit-values of 0 or 1, determined by comparing the photodiode output voltage to a threshold voltage. This uncorrelated randomness is essential for generating high-quality random numbers in our QRNG. The outputs from each channel, A and B, are processed by a field-programmable gate array (FPGA), which executes an A ⊕ B operation to produce the final random bit-stream.

**Fig. 2:**
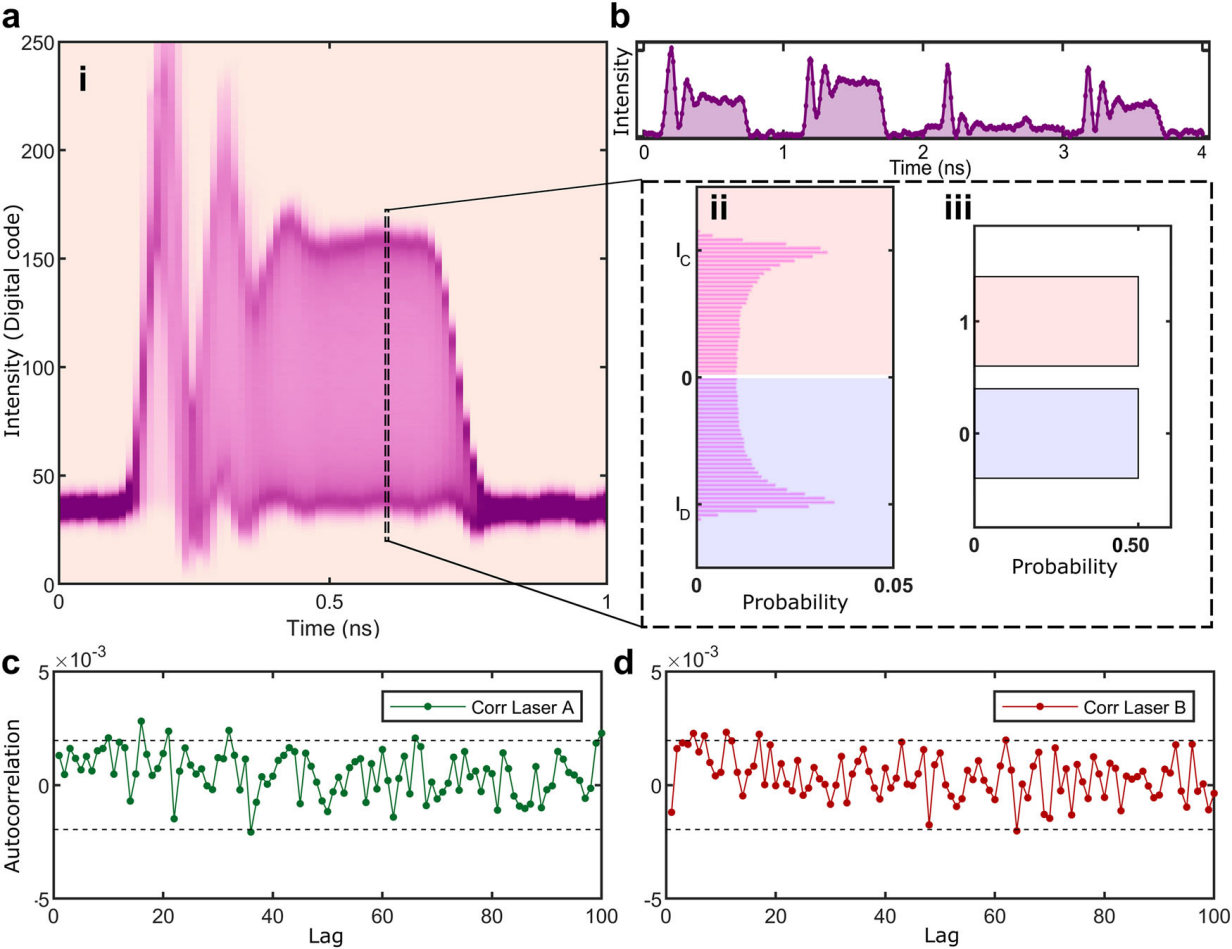
Laser interference pattern with noise characterization and byte uniformity. **a**(i) The waveform density plot of a single laser gain-switched at 1 GHz. **a**(ii) The area enclosed within the dashed rectangle denotes the sampled time bin. The distribution of the measured intensity values follows an arcsine distribution, with visibility between *I*_*C*_ and *I*_*D*_. **a**(iii) For an appropriately chosen comparator value, which evaluates all events above a given threshold as binary 1 and all those below the threshold as binary 0, we find an even distribution of each bit value (0,1). **b** PD signal after the AMZI up to 4 ns, a subset of the signals used to construct (**a**). **c**, **d** The auto-correlation of byte values recorded up to 100 lags of laser A and laser B, respectively, for a sample size of 10^6^ and 95% confidence interval.

First, we characterize our device by measuring the output of the PDs with an oscilloscope. In Fig. 2a(i–iii), we show the waveform probability density when the laser is pulsed at a repetition rate of 1 GHz, constructed from a 10^6^ pulse-long waveform. The dashed line shows a slice through the waveform probability density, revealing the arcsine distribution. We place a threshold intensity bin level (white line for illustrative purposes) in Fig. 2a(ii) in the central bin of the distribution, which after integrating the red and blue regions result in an unbiased output from the waveforms. In Fig. 2b, the intensity received on the photodiode is shown, where each data point is formed from the interference of a pulse with a pulse from the previous clock cycle. In Fig. 2c, d, we show the autocorrelation of the byte values up to a lag of 100 clock cycles for the output of laser A and B. Here we see low correlation between optical pulses indicating that phase randomization through optical gain-switching has been achieved.

### NIST statistical test suite

**Fig. 3:**
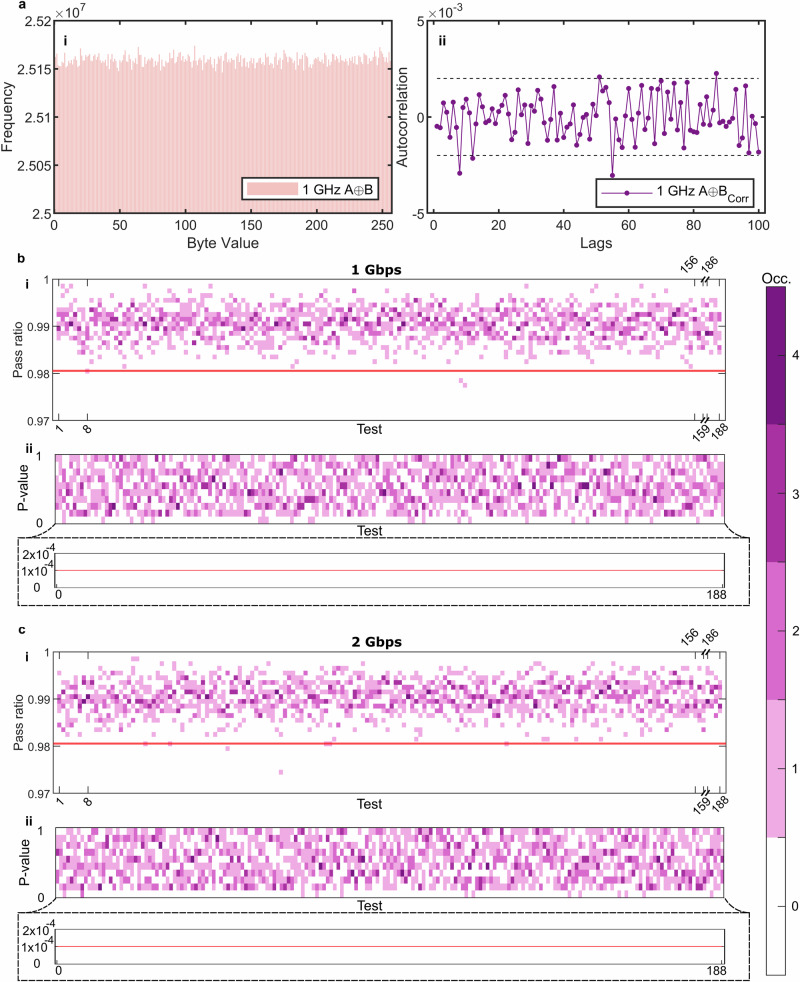
NIST test results. 1 Gbit of XOR output from the QRNG operating at 1 Gbps was analyzed: **a**(i) shows the uniformity of byte values between 0–255. **a**(ii) shows the autocorrelation for up to 100 lags with confidence bounds at 95% on a block size of 10^6^. **b** displays the results of NIST test suite on 9 separate files when using the XOR output at 1 Gbps from the QRNG, the proportion of passes (i) on each test is plot. Columns correspond to the tests from 1 to 159 and from 186 to 188. Columns from 9 to 156 correspond to the Non-overlapping Template test. The solid red line is the pass threshold (0.980561). Tests from 160 to 185 are not reported because they feature a different threshold for each of the files (we report a total of four values slightly below threshold out of a total of 1692, one of which is not shown in the plot). **b**(ii) shows the *p* value distribution aggregated over the same nine files. The upper panel bins 0 ≤ *p* ≤ 1 in steps of 0.1; the inset beneath resolves the critical region 0 ≤ *p* ≤ 2 × 10^−4^ with a 1 × 10^−5^ bin width. The horizontal red line marks the significance level of 1 × 10^−4^ used by the NIST suite. In this data no *p* value falls below the critical value. **c** shows the same analysis but at 2 Gbps. In (i) we report a total of eight values slightly below threshold (out of a total of 1692), one of which is not shown in the plot. **c**(ii) *p* value histograms for 2 Gbps, formatted in the same manner as (**b**(ii)); no *p* value violates the 1 × 10^−4^ criterion. The color-grade encodes the number of occurrences of values that fall within each bin range.

Following the characterization we study the digital output of the clocked comparators. We generate a sequence of 0 s and 1 s from the phase randomized pulses at up to 2 Gbps. Here, we note that the pulses are separated by two clock cycles in our 1 ns AMZIs. The FPGA performs an A ⊕ B operation on the binary sequences which is streamed as UDP packets via ethernet (SFP 10G) to a computer for analysis. In Fig. 3a(i), the byte uniformity of the QRNG is shown for a rep. rate of 1 GHz. Analysis of a 1 GB (gigabyte) sample of the random output shows that the bytes transmitted from our device are uniformly distributed. In Fig. 3a(ii), we show the autocorrelation between the bit-values up to a lag of 100 clock cycles for a subset of the data (10^6^ samples). Importantly, we observe no significant correlations for the sample length considered.

A hallmark of random numbers is their ability to pass stringent statistical tests^28,29^. The National Institute of Standards and Technology (NIST) provides a suite of certifiable randomness tests, which we applied to 1 Gbit samples generated by our device. The result of these tests performed on the outputs at 1 Gbps and 2 Gbps are displayed in Fig. 3b, c. The tests were performed by dividing the 1 Gbit sample into *L* = 1000 smaller substrings and running each test *L* times. According to the recommendations of the suite, the tests have a significance level of 1 × 10^−2^. Each test outputs a *p* value: if it is ≥1 × 10^−2^ the test is passed. For the entire set of *L* tests, a second-order analysis is conducted by calculating a *χ*^2^ test statistic for the distribution of the *L* *p* values across ten bins. The *χ*^2^ statistic is assigned a *p* value. A test on the entire input data is considered successful if approximately 98% of the tested substring pass and if the *p* value assigned to the uniformity of *p* values for each statistical test is ≥10^−4^.

In Fig. 3b, c, we show the results of 9 sets of tests taken over a ~14-h measurement period. A 1 Gbit sample is recorded and then processed 9 times over this period, for each acquisition the proportion and *p* values are shown for each test. The acquisition of each 1 Gbit sample automatically takes place after the preceding sample has been processed. The processing time for each sample is ~1.5 h. In Fig 3b(i-ii), we show the 1 Gbps results, while in Fig. 3c(i-ii) we show 2 Gbps. In Fig. 3b(i), c(i) the critical value for the pass ratio (marked by a red line) was 0.980561 for the dataset input, indicating that the test has passed for the set of *L* substrings. The pass rate for the “random excursion (variant)” test varies between each acquisition, so we omit the data from the heatmap for clarity. The *x*-axis indicates the test type which are detailed in ref. ^29^. In Fig. 3b(ii), c(ii), the *p* values across the 9 runs are binned for each test and the occurrence frequency of values that lie within each bin are shown. The upper panel bins 0 ≤ *p* ≤ 1 in steps of 0.1 to highlight uniformity; the inset beneath resolves the critical region 0 ≤ *p* ≤ 2 × 10^−4^ with a 1 × 10^−5^ bin width. The horizontal red line marks the significance level of 1 × 10^−4^ used by the NIST suite. In the Supplementary Material we show the results of a longer term study at 1 Gbps. The data from Fig. 3b, c indicate that our device is operating as expected and that testing the output binary sequences using the NIST suite does not reveal any concerning behavior or statistically relevant deviations from the expected distribution.

### Free-space QKD using QRNG seed

Having demonstrated that our QRNG can output random bits at up to 2 Gbps, its random output is then used to seed a BB84 transmitter as part of a free space QKD system.

We implement the T12 efficient decoy-state BB84 protocol over a lab-based free-space experiment. This protocol utilizes decoy states to protect against eavesdropping attacks^30,31^, making it a robust choice for satellite-based quantum communications. Using eight vertical-cavity surface-emitting lasers (VCSELs) at 850 nm, we encode in four polarization states: horizontal, vertical, diagonal, and anti-diagonal polarizations, which represent two mutually unbiased bases, Z and X. The biases in the selection probabilities (p(Z) > p(X)) are set to maximize the number of events used for key generation (Z) while ensuring a sufficient number of events in the minority basis (X) for error estimation. A schematic of the experiment can be found in the Supplementary Material and follows from our previous work^20^.

**Fig. 4:**
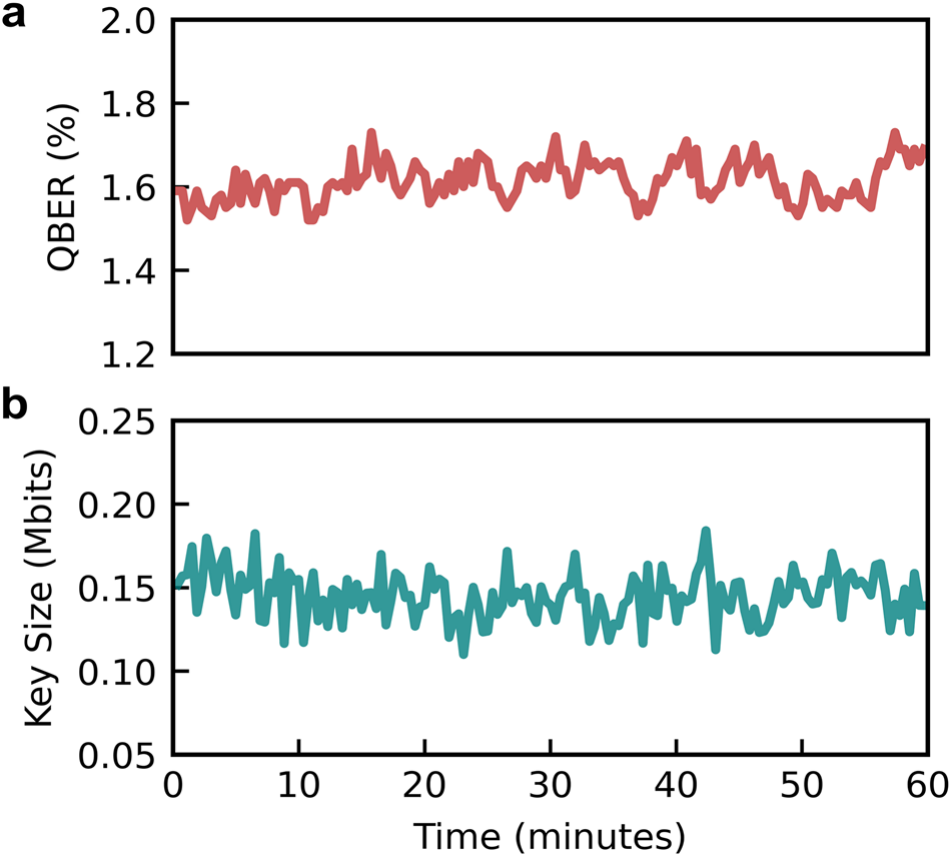
QKD results. **a** shows the QBER of our system over a period of 60 min. **b** shows the key size of each generated key at an average key rate of 0.013 Mbps, under a fixed channel loss of 20 dB.

We stream our QRNG into the QKD system at 1 and 2 Gbps. The QKD system uses randomness expansion to generate random binary sequences at 13 Gbps for the QKD protocol operating at 1 GHz. Fig. 4 shows the results for the highest streaming rate of 2 Gbps. We monitor QBER (a) and key size (b) of the QKD system for 1-h acquisitions to show the integration of our system. Increasing the rate of the QRNG enables the seed that is used as the input to the randomness expansion to be refreshed more often, increasing the security of the system. Moreover, the real-time streaming of our QRNG, combined with the QKD system’s ability to consume random bits without storing them, mitigates the risk of an adversary gaining knowledge of the secure key. In our previous study^20^ we show the same QRNG architecture can be used to seed the free-space QKD system at 1 Gbps in an emulated overpass of satellite-to-ground QKD.

### Discussion

The raw outputs of all QRNGs include contributions other than the quantum noise they are designed to exploit, that is, classical noise and hardware non-idealities. Post-processing, such as Toeplitz hashing, is commonly used to extract uniformly distributed, unpredictable random numbers from the raw output^32^. This post-processing consumes a large amount of power. Through testing of a comparable system^33^, we find a 25% increase in the power consumed by the device compared to when the hashing algorithm is switched off. For applications where keeping the power consumption of the device low is critical we must look to other post-processing methods. It is important to note that while this QRNG architecture can be integrated with minimal additional power requirements—comprising 7.93 W for the FPGA chipset and associated opto-electronic PCB—our experimental setup employs an FPGA evaluation board for system testing. In practical implementations, such as within a QKD system that already incorporates an FPGA chipset, the QRNG logic could be directly embedded into the existing programmable fabric. The evaluation board used in our experiments includes numerous high-power peripherals (e.g., DDR4 RAM, QSPI flash, Ethernet PHY) that were not utilized during testing. Consequently, the total power consumption measured—14.7 W—reflects the overhead introduced by these unused components. A detailed breakdown of the system’s power consumption is provided in the Supplementary Material.

Modern quantum key distribution (QKD) systems operate at gigahertz (GHz) clock rates, consuming significant amounts of random bits per clock cycle. Specifically, our QKD system requires 13 random bits per cycle, necessitating a 6.5-fold seed expansion from the bits provided by the quantum random number generator (QRNG). It should be noted that the required expansion of bits could be reduced to 1.5x by implementing the T12 protocol with optimally designed Huffman-like coding^34^, whereby the protocol can be encoded with approximately 3 bits on average per clock cycle. To enable direct encoding of the protocol at 1 GHz, without need for expansion of the bits, we could further enhance the rate of our existing QRNG, by increasing its speed or by multiplexing additional devices. Please refer to the Supplementary Material for results demonstrating our device operating at 4 Gbps.

In the QRNG presented in this paper, the 1-bit ADC outputs an approximately uniform distribution by placing the comparator threshold at the center of the symmetric arcsine intensity profile. This technique offers the advantage that the intensity distribution is at a local minimum and any fluctuations in the signal lead to very small changes in the bias of the output bits. Performing A ⊕ B operation further reduces any bias at the output, forming strings of uniformly distributed random bits. This provides a low-power route to removing unwanted bias in the output. Note, this method can become problematic if significant correlations are present within the raw bits^35^.

The information-theoretic security of quantum key distribution (QKD) can only be ensured if the randomness source for the encoding is generated by a quantum process with inherent non-deterministic nature. While satellite-assisted quantum communication is a relatively new area of development, many missions are planned to establish a global quantum-safe network. Existing satellite-to-ground QKD systems operate at a low clock rate and use physical random number generators to seed the QKD system. With real-time processing of QKD data, facilitated by satellite-to-ground laser communication^36^, the clock rate of the transmitter can be increased to enhance throughput. Our solution for a low-SWaP QRNG could enable high-speed randomness seeding for spaceborne quantum transmitters.

In summary, we have demonstrated and characterized a fast, low-power 2 Gbps QRNG using clocked comparators. We showed that the XOR technique is robust when applied to the two random binary sequences generated from our phase noise laser sources. Finally, we successfully performed a real-time QKD protocol with the QRNG seeding the transmitter, highlighting its practical application and effectiveness in secure quantum communication.

## Methods

### Architecture and operation

The low-SWaP QRNG PCB was designed to operate within a <10W power constraint^18^. The PCB design fits into a 13 × 18 cm form factor and contains all the necessary driving and readout electronics, including two laser diode (LD) drivers, a thermoelectric cooler (TEC), an analog-to-digital converter (ADC), a photodiode (PD) bias circuit, and a digital-to-analog converter (DAC). With satellite operation in mind, the QRNG operation is robust over time, demonstrating minimal output bias drift over 8 days of continuous operation at 1 Gbps with no user intervention. Over this period 〈Bit bias〉 = 0.500001 ± 4.965 × 10^−7^ (SE), with the average within the 99% confidence interval of the ideal value 0.5, see the Supplementary Material for more details.

The FPGA is used to both transmit and receive data to/from the PCB, driving the laser diodes and collecting the random bits generated by the photodiodes and comparators. The FPGA transmits and receives data at 8 GSa/s, providing a minimum electrical pulse width of 125 ps. The comparator is clocked such that on a rising edge of a pulse provided by the FPGA the electrical output of the photodiode is compared to a threshold value, chosen to balance the number of 0s and 1s produced at the output of the comparator. The gating of the comparator is performed at 4 GHz and then sampled by the FPGA, depending on the driving rate of the lasers and corresponding AMZI. The precise temporal position that the comparator samples within the optical pulse train is selected by delaying the gating signal of the comparator.

We use fully-passive silica AMZIs, fabricated using an ion exchange process, providing ultra-low insertion losses and calibrated to produce high visibility interference between the short and long arms. Light is split equally by a 50:50 coupler into two output waveguides, a short and a long path, that recombine at an output 50:50 coupler. The long path features a 1 ns delay line whose excess propagation loss is compensated for using a loss element in the short path. This approach avoids tunable couplers often used in alternative approaches, saving up to 0.8 W power consumption in total.

## Supplementary information

Supplementary Information

## Data Availability

The data that support the findings of this study are available from Toshiba CRL Europe under reasonable request.
