# Supplementary material for: A 2-Gbps low-SWaP quantum random number generator with photonic integrated circuits for satellite applications
Source: npj Quantum Inf. 2025 Sep 26;11(1):153. doi: 10.1038/s41534-025-01100-2 (PMC12474547; doi:10.1038/s41534-025-01100-2)
Supplement: Supplementary file 1 — Supplementary Information [file 41534_2025_1100_MOESM1_ESM.pdf]

Supplementary Material: A 2-Gbps Low-SWaP Quantum Random Number Generator with Photonic Integrated Circuits for Satellite Applications

Oliver M. Crampton,[1,2] Toby J. Dowling,[1,*] Thomas Roger,[1,†] Peter R. Smith,[1] James F. Dynes,[1] Matthew S. Winnel,[1] Davide G. Marangon,[1] Mirko Sanzaro,[1] Ravinder Singh,[1] Chithrabhanu Perumangatt,[1] Joseph A. Dolphin,[1] Taofiq K. Paraiso,[1] and Andrew J. Shields[1]

[1] *Toshiba Europe Limited, 208 Cambridge Science Park Milton Rd, Milton, Cambridge*

[2] *School of Engineering and Physical Sciences, Heriot-Watt University, Edinburgh*

[*] toby.dowling@toshiba.eu

[†] thomas.roger@toshiba.eu

SUPPLEMENTARY 1: AUTOCORRELATION

One of the most important tests to evaluate the degree of phase-randomization is the autocorrelation between subsequent interferences on a photodiode. We collect the output of the asymmetric Mach-Zender interferometers (AMZIs) by the schematic in Fig. 1. The driving current and modulating signal are set using an FPGA to achieve weak coherent pulses that, when interfered with a following pulse, produce the random intensity shown in the Main Text. Here we show additional waveforms collected at 2 and 4 GHz laser repetition rate, showing that the lasers in the device can work at this high rate – Fig. 2a-b. When the laser is set to 4 GHz, we have 20 sampling points per pulse, leading to a very narrow temporal window to see the phase-randomised pulses. The interference no longer has a long steady-state region at 2 GHz and 4 GHz clock rates.

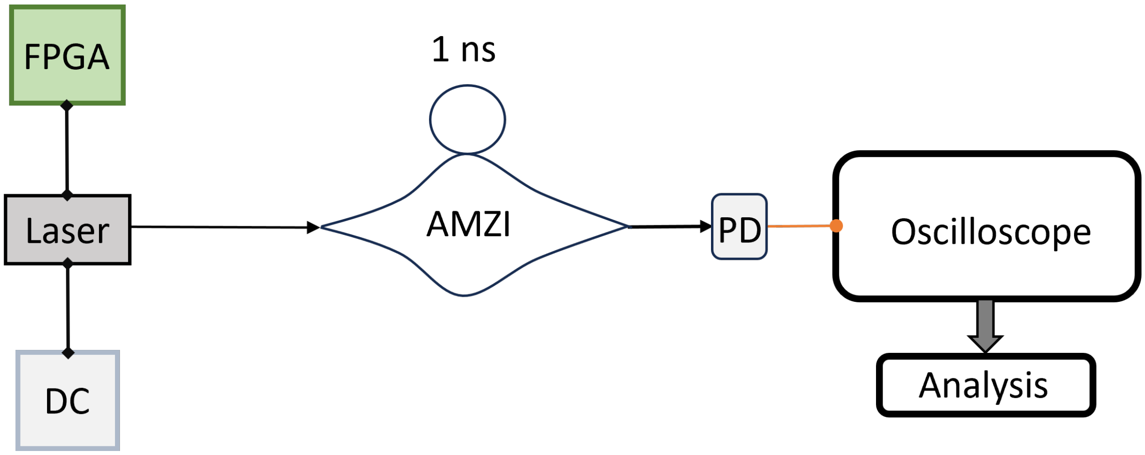

Supplementary Figure 1. **Waveform data collection.** The laser diode is gain-switched using a clock signal from the FPGA. The pulses pass through a 1 ns delay AMZI and the intensity distribution is recorded on a fast photodiode with an oscilloscope.

The autocorrelation of the waveform from laser A are shown at 2 GHz and 4 GHz

in Fig. 2c-d. We draw the same conclusion from the 2 GHz results as those presented in the Main Text, that we see no correlations at early lags – using a 95 % confidence interval on a lag size of 100 with a sample size of one-million pulses. However, at 4 GHz we clearly see the correlation emerge at the first lag, suggesting that full phase randomization has not been achieved. The possible reasons for this could be: cavity emptying effects due to there not being enough time allowed for carriers to exit the cavity before pulsing, or spectral broadening effects that degrade the interference.

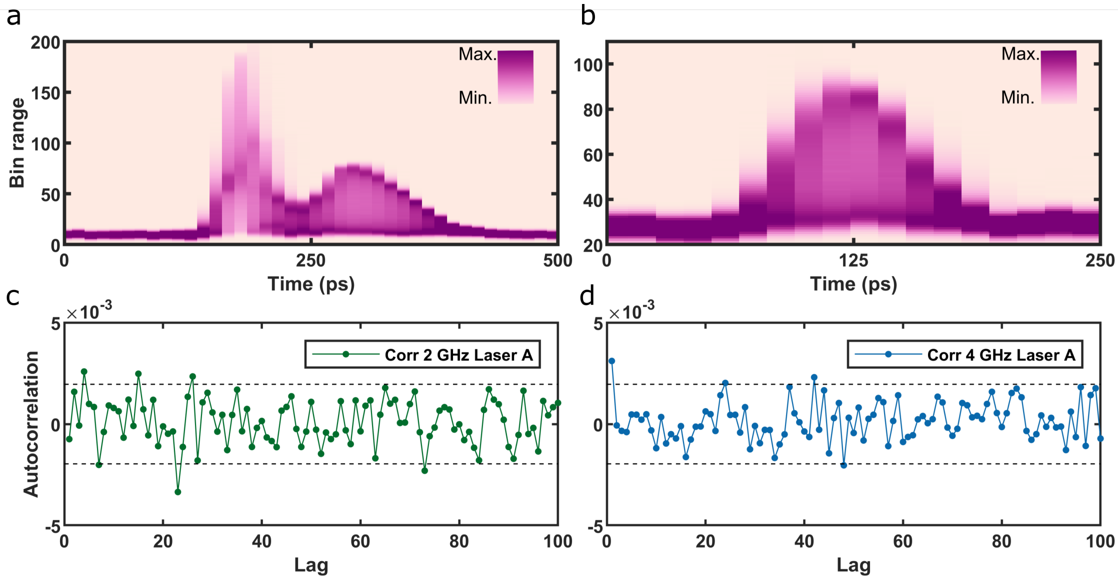

Supplementary Figure 2. **Waveform and autocorrlation from interference measurements at 2 GHz and 4 GHz.** **a**, waveform of the interference measurement at 2 GHz clock rate on laser A. **b**, waveform of the interference measurement at 4 GHz clock rate on laser A. **c**, autocorrelation of the bit-stream from a sample point on (a). **d**, autocorrelation of the byte values recorded on the oscilloscope from a sample point on (b).

SUPPLEMENTARY 2: NIST SUITES RESULTS FOR 4 GBPS

In this study, we use an open source test suite provided by the National Institute for Standards and Technology, NIST, to validate the uniformity and statistical randomness of the numbers generated from our device. As shown in the Main Text, measurements performed at 1 and 2 Gbps clock speeds we have a statistically consistent pass rate when considering multiple measurements of binary sequences. The NIST results for the XOR output at 4 Gbps are shown in Fig. 3. Here, most tests exhibit high pass ratios and and uniformly distributed p values. However, this setting also shows notable failures— most prominently, the Block Frequency test, which failed in all 53 acquisitions.

SUPPLEMENTARY 3: DECOY STATE QKD MEASUREMENT

In the Main Text, we present the key size and quantum bit error rate (QBER) from a 2 Gbps $A \oplus B$ operational mode. In Fig. 4, we show the experimental setup. The QRNG is used to seed a quantum transmitter, which encodes 4 polarization states using three different levels: signal, decoy and vacuum. The transmitter FPGA communicates with the receiver FPGA classically at C-band wavelengths 1530 and 1550 nm. Eight 850 nm VCSELs are combined using a PM fiber coupled combiner, to produce the 4 polarization states. The quantum signal is multiplexed with the classical signal using a dichroic mirror.

At the receiver, a dichroic mirror is used to separate the classical and quantum signals, and a 90:10 beam-splitter passively selects the measurement basis, a half-wave plate is used to rotate the basis to diagonal for one pair of detectors. The counts on four single photon detectors are time-tagged by the Rx FPGA which performs sifting via the classical channel.

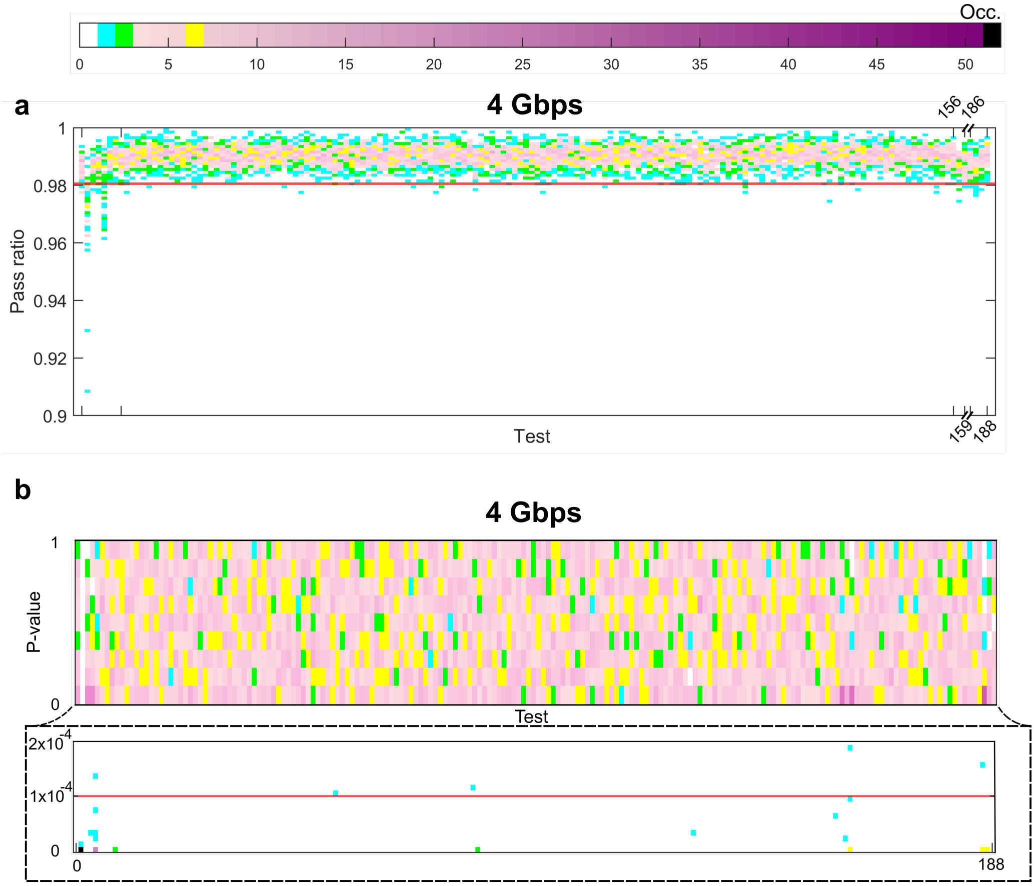

Supplementary Figure 3. **NIST Test Results at 4 Gbps.** (**a**) displays the results of the NIST test-suite on 53 separate files using the XOR output from the QRNG at 4 Gbps; the proportion of passes on each test is plot. Columns correspond to the tests from 1 to 159 and from 186 to 188. Columns from 9 to 156 correspond to the Non-Overlapping Template test. The solid red line is the pass threshold (0.980561). Tests from 160 to 185 are not reported because they feature a different threshold for each of the files. At 4 Gbps there are critical failures of test 2 (Block-Frequency test) and test 5 (Runs test). Out of a total of 9 964 tests we record 150 failures, with a total of 4 not shown. (**b**) P value distribution aggregated over the same nine files. The upper panel bins $0 \leq p \leq 1$ in steps of 0.1; the inset beneath resolves the critical region $0 \leq p \leq 2 \times 10^{-4}$ with a 1×10^{-5} bin width. The horizontal red line marks the significance level of 1×10^{-4} used by the NIST suite. The 53 p values per test highlight the distribution of p values observed on this data-set. We record 104 failures, with the most prominent being 100 % failure on the Block-Frequency test. The color-grade encodes the number of occurrences of values that fall within each bin range.

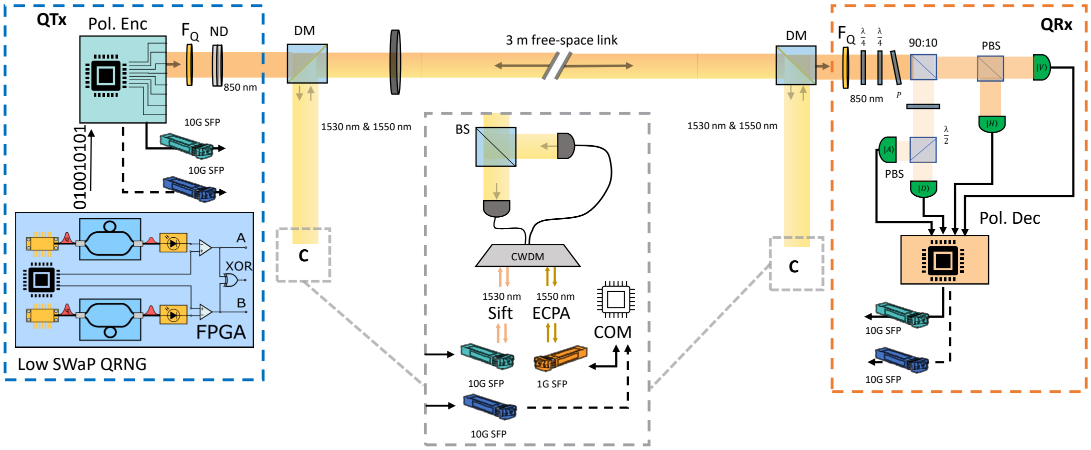

Supplementary Figure 4. **Decoy-state free-space QKD protocol with low SWaP QRNG seeding the transmitter moddule.** Two DMs, are used to multiplex/demultiplex the bidirectional classical communications lasers (C), and the quantum signal at the QTx and QRx. The polarization decoder at the QRx consists of a series of wave plates ($\lambda/4$, Q) to align the decoding reference frame to that of the transmitter, and the measurement basis is passively selected by a 90:10 BS (90:10). The four polarization states are measured by four single-photon detectors via PBSs with a half-wave plate ($\lambda/2$) used to rotate one of the detector pairs into the diagonal, anti-diagonal polarization (minority) basis. The QTx is seeded by a QRNG that is used to generate the quantum key in real time. The quantum signals are optically filtered (F_Q) to increase the signal-to-noise ratio. A fixed ND filter was used for this experiment giving a total channel loss of approximately 20 dB. Classical data are transmitted by two SFP transceivers, including the sifting traffic, EC, and PA. The transmit/receive optical signals for the classical laser communications are combined to the same beamline via a BS. Secure keys are transmitted to a local computer (COM) and are stored locally to each subsystem (QTx, QRx).

SUPPLEMENTARY 4: LONG-TERM BIT BIAS

We monitored the bit bias over a block size of 1048576, which is shown in Fig. 5, logging the bias value 967032 times over approximately 8 days. The bias is stable over this period, observing only 1.02% of values outside the confidence bound. Over this period $\langle \text{Bit bias} \rangle = 0.500001 \pm 4.965 \times 10^{-7}$ (SE), with the average within the 99% confidence interval of the ideal value 0.5.

We also periodically collected data and performed NIST tests on the output during this long term monitoring period, for a total of 129 files. The results of which are shown in Fig. 6.

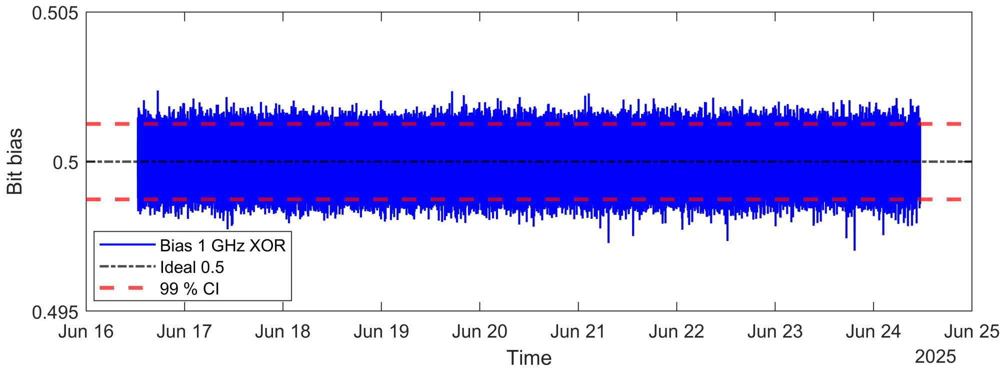

Supplementary Figure 5. **Bit bias log for 1 Gbps XOR output.** Bit bias recorded on a block size of 1048576. We log 967032 values over an approximately 8 days. The NIST test results Fig. 6 were recorded intermittently over the same time interval of this data collection window.

SUPPLEMENTARY 5: POWER CONSUMPTION ANALYSIS

The QRNG architecture comprises three primary components: (1) the FPGA chipset, (2) the evaluation board that hosts the chipset along with various peripheral

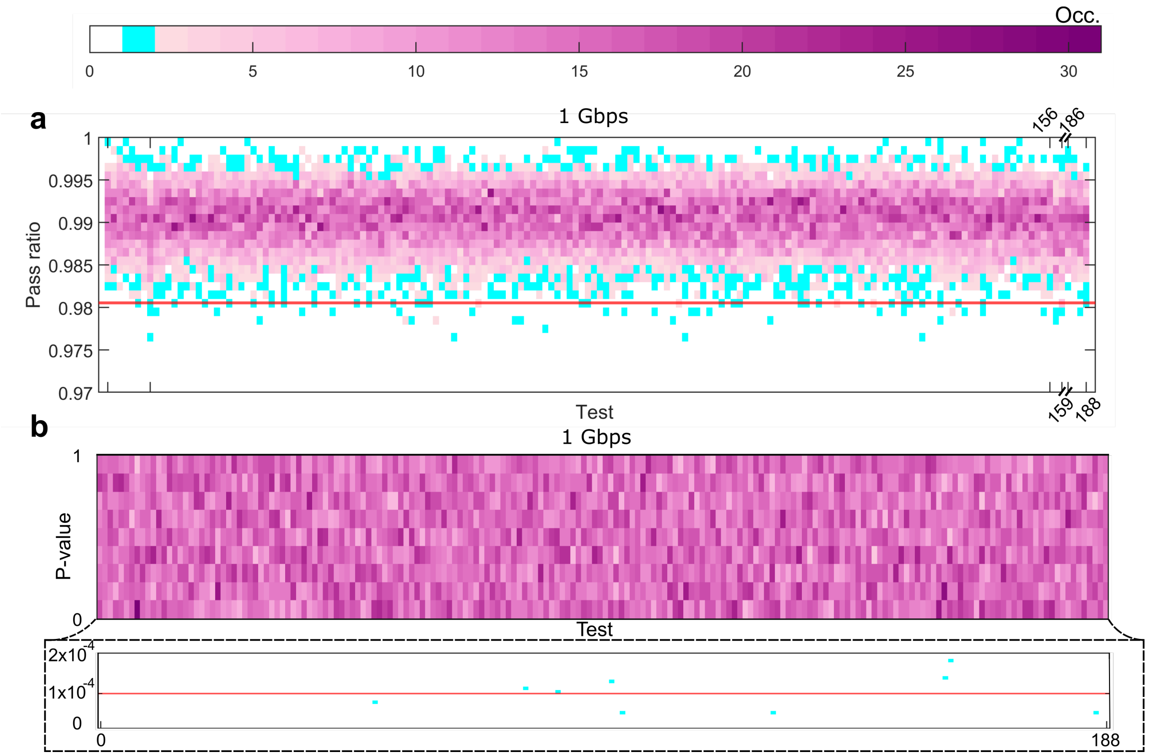

Supplementary Figure 6. **NIST Test Results at 1 Gbps over 8 days.** (**a**) displays the results of the NIST test suite on 129 separate files from the XOR output from the QRNG at 1Gbps; the proportion of passes on each test is plot. Columns correspond to the tests from 1 to 159 and from 186 to 188. Columns from 9 to 156 correspond to the Non-Overlapping Template test. The solid red line is the pass threshold (0.980561). Tests from 160 to 185 are not reported because they feature a different threshold for each of the files. At 1 Gbps there are critical failures of test 2 (Block-Frequency test) and test 5 (Runs test). Out of a total of 24 252 tests we record 110 values slightly below the pass threshold, 25 of which are not shown. (**b**) P value distribution aggregated over the same 129 files. The upper panel bins $0 \leq p \leq 1$ in steps of 0.1; the inset beneath resolves the critical region $0 \leq p \leq 2 \times 10^{-4}$ with a 1×10^{-5} bin width. The horizontal red line marks the significance level of 1×10^{-4} used by the NIST suite. The 129 p values per test highlight the uniformity of the p value distribution across this large data set. We see a failure on four individual tests: three Non-Overlapping Template instances and one Serial. The color-grade encodes the number of occurrences of values that fall within each bin range. The data was acquired over the same time interval as Fig. 5

interfaces, and (3) an optoelectronics PCB that integrates the laser diodes (LDs), photodiodes (PDs), and associated analog-digital interface circuitry.

**Resource Type**	**Used**	**Available**	**Utilization (%)**
LUT	13,292	242,400	5.48
LUTRAM	1,830	112,800	1.62
FF	20,458	484,800	4.22
BRAM	21	600	3.50
IO	20	520	3.85
GT	6	20	30.00
BUFG	12	480	2.50
MMCM	1	10	10.00

Supplementary Table I. FPGA Resource Utilization Summary

Power consumption for each of these components was monitored over a continuous 15-h operational period. The results are presented in Fig. 7 - Fig. 9.

Fig. 7 illustrates the power consumption of the FPGA chipset, measured across multiple power rails that supply distinct functional blocks within the FPGA. The current on each rail was inferred by measuring the voltage drop across precision shunt resistors placed in series with the supply lines. These measurements were sampled every 30 seconds, and the total power consumption was computed by summing the contributions from all monitored rails.

Fig. 8 presents the power profile of the evaluation board used in the experiments. This includes the power drawn by the FPGA chipset itself, as well as additional board-level components. It is important to note that many of these peripherals are not utilized by the QRNG architecture and thus represent overhead not intrinsic to the QRNG design.

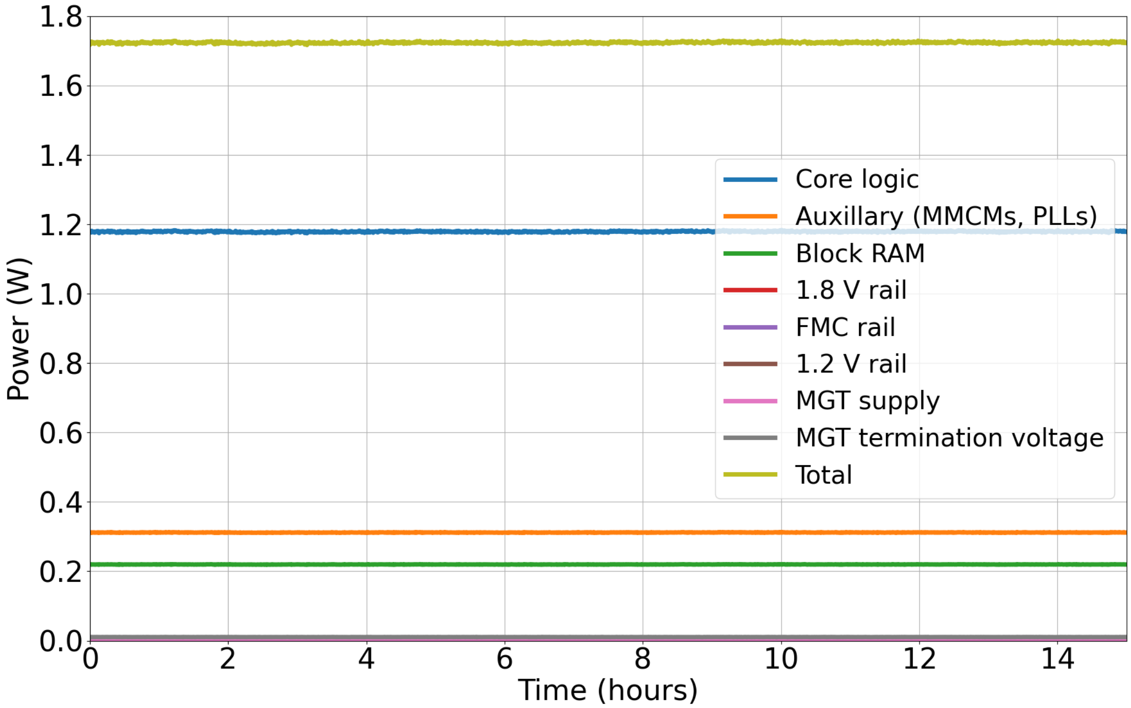

Supplementary Figure 7. **Monitor rails power consumption over time.** ADCs measured the voltage drop across shunt resistors on rails that supply various parts of the chipset. The total power summed over all rails shows a stable power draw of 1.72 W over the 15-h measurement. The measurements of power were periodically measured at 30 second intervals.

Fig. 9 shows the power consumption of the optoelectronics PCB, which includes the active optical components and their interface circuitry.

Although the evaluation board exhibits a relatively high power draw, this is largely attributable to unused subsystems. In a practical deployment, the QRNG would be integrated directly onto the FPGA chipset already present in the QKD system, thereby eliminating the need for a separate evaluation board. Under this assumption, the additional power overhead introduced by the QRNG can be attributed solely to

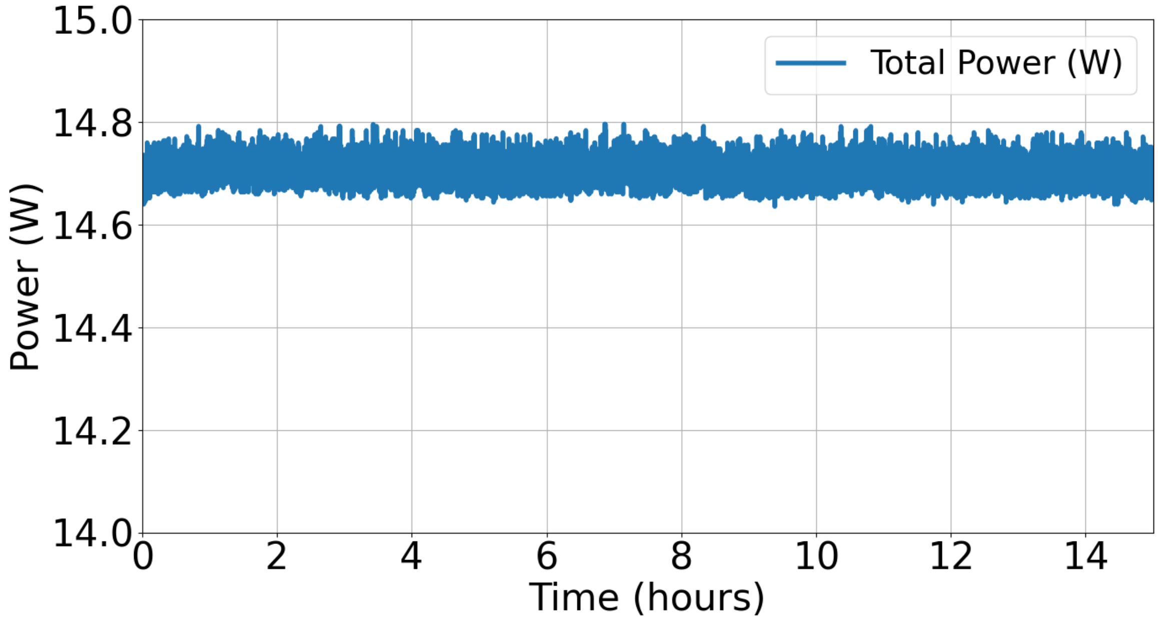

Supplementary Figure 8. **FPGA chipset power consumption over time.** 12 V supply line is used to power the various ICs (DC-DC converters, laser drivers, gated-comparators, i2c multiplexers etc.). The power was read every 5 seconds.

the FPGA chipset and the optoelectronics PCB.

Table I summarizes the FPGA resource utilization for the implemented QRNG design. The results indicate that the design is sufficiently lightweight to be integrated into the existing FPGA infrastructure of a QKD system.

The average power consumption attributable to the QRNG components (FPGA chipset and optoelectronics PCB) over the 15-h measurement period is 7.973 ± 0.005 W. When including the evaluation board, the total system power draw increases to 20.953 ± 0.030 W.

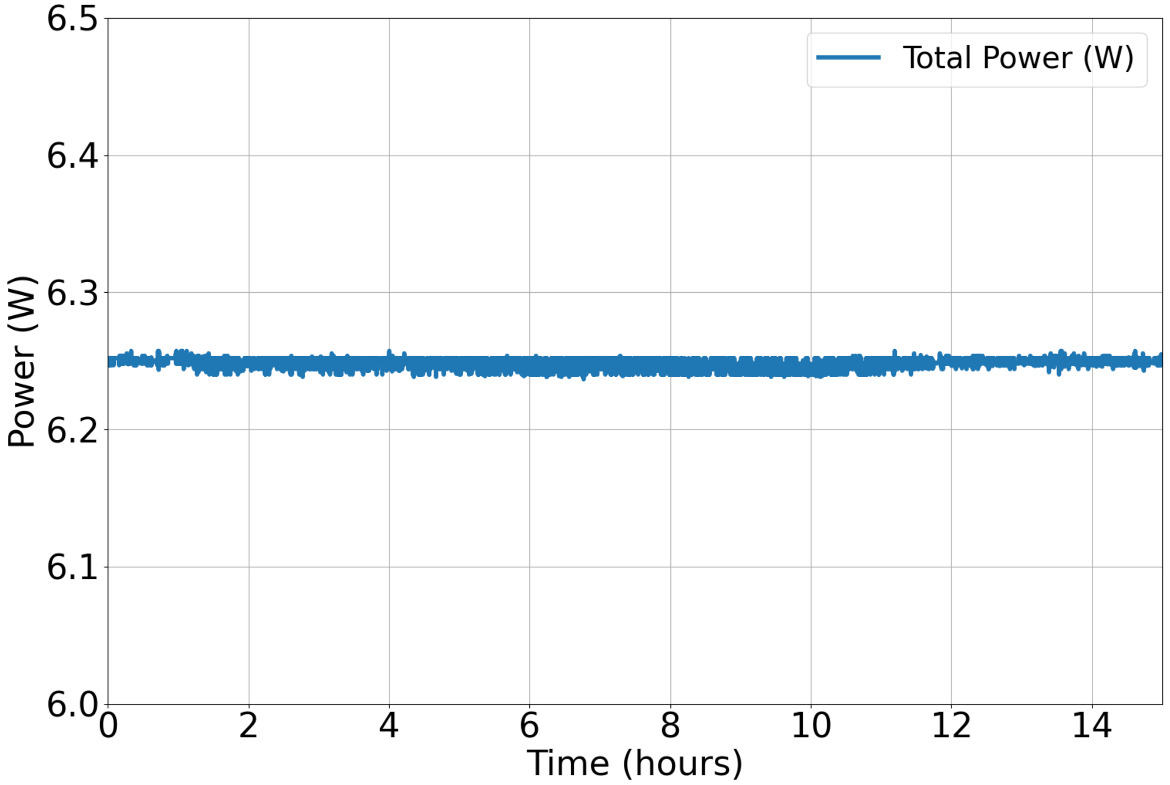

Supplementary Figure 9. **PCB power consumption over time.** The power drawn over the 15-h measurement was stable around 14.75 W. Note that much of this power is associated with peripherals that were not used in the QRNG architecture such as DDR4 RAM, HDMI interface, QSPI memory, Ethernet PHY. The power was read from the Power Supply Unit (PSU) every 5 seconds.

DATA AVAILABILITY

The data that support the findings of this study are available from Toshiba CRL Europe under reasonable request.

ACKNOWLEDGEMENTS

AUTHOR CONTRIBUTIONS

TR, TJD, OC and DM wrote the supplementary material with input from all the authors.

COMPETING INTERESTS

The authors declare no competing interests.
